# Evaluating Early Asymptomatic Postoperative Sinus Bradycardia Following Sleeve Gastrectomy: A Preliminary Observational Study

**DOI:** 10.1007/s11695-025-08173-0

**Published:** 2025-08-26

**Authors:** Ahmed Abokhozima, Hassan El-Masry, Mohamed H. Zidan, Ahmed Abo Elmagd, Hashem Altabbaa, Moutaz Abdel Mohsen Ghandour, Hisham Hanaa, Aliaa Selim, Mohammed Alokl, Moustafa Madkour

**Affiliations:** 1 Faculty of Medicine, Alexandria University, Alexandria, Egypt; 2 Anesthesia and Surgical Intensive Care, Faculty of Medicine, Alexandria University, Alexandria, Egypt; 3 Anesthesia Department, Alexandria University Students Hospital, Alexandria, Egypt; 4 Cardiology Department, Faculty of Medicine, Alexandria University, Alexandria, Egypt; 5 Al Basheer Hospital, Amman, Jordan

**Keywords:** Early postoperative sinus bradycardia, Sleeve gastrectomy, Autonomic dysfunction, Heart rate recovery, Sinus bradycardia after bariatric surgery

## Abstract

**Introduction:**

Postoperative bradycardia, particularly in the early postoperative phase, is a potential complication following bariatric surgeries such as sleeve gastrectomy (SG). While various types of bradycardias have been documented as complications, early-onset sinus bradycardia has received limited attention. This case series specifically investigates the incidence and progression of early postoperative sinus bradycardia in patients undergoing laparoscopic SG, excluding those with hiatal hernias or cardiovascular comorbidities.

**Methods:**

We conducted a retrospective series of patients aged 18 to 50 years with morbid obesity (BMI ≥ 35 kg/m2) who underwent laparoscopic SG from January 2016 to March 2023. All patients were monitored for postoperative bradycardia, defined as heart rates below 60 beats per minute (bpm), and were evaluated with electrocardiograms (ECG) and echocardiograms (ECHO). Data were analyzed using descriptive statistics and R software version 4.3.1.

**Results:**

The study included eight patients with a mean age of 34.88 ± 9.05 years and a mean BMI of 44.25 ± 3.62. Postoperative heart rates showed an initial increase to 79.6 ± 4.8 bpm, followed by a gradual decline, reaching 50 ± 3.17 bpm by discharge. Heart rates dropped significantly after 12 h, stabilizing by 24 h. The mean time to return to normal heart rate was 14 ± 4.36 days. All patients had routine preoperative ECG and ECHO, and no significant cardiac abnormalities were noted postoperatively.

**Conclusion:**

This case series underscores the occurrence of early postoperative sinus bradycardia in SG patients, a phenomenon that is underexplored in the literature. Future studies should investigate the pathophysiology of this condition and evaluate its clinical implications for postoperative care.

## Introduction

Cardiac arrhythmia is a potentially life-threatening condition that can manifest with various symptoms. Postoperative bradycardia can present after abdominal surgeries, resulting from excessive visceral manipulation. Hiatal hernia is a common surgical condition that has been associated with cardiac arrhythmias, including sinus bradycardia and complete heart block. This is due to the compressive effects on the heart or vagal stimulation caused by the hernia [1, 2].

Although obesity is a well-known risk factor for hiatal hernia, bariatric procedures like sleeve gastrectomies (SG) have been associated with postoperative arrhythmias without any connection to hiatal hernias. Furthermore, postoperative sinus bradycardia is usually seen in the late postoperative period and is attributed to massive weight loss. Early postoperative sinus bradycardia has not yet gained a major highlight in the literature and has only been reported once [3]. In this observational study, we examined the incidence of early postoperative bradycardia in the immediate postoperative period.

## Patients and Methods

This study presents a unifocal retrospective case series derived from a single surgeon’s well-designed, systematically collated electronic medical records. The study has been reported in line with the PROCESS guidelines [4]. Ethical approval was granted by the Institutional Review Board (IRB) of the Faculty of Medicine, Alexandria University (IRB No. 00012098). All procedures complied with the ethical standards of the institutional research committee and the 1964 Helsinki Declaration. The cohort comprised individuals aged between 18 and 50 years, each diagnosed with morbid obesity with a BMI of 35 kg/m^2^ who had undergone laparoscopic sleeve gastrectomy (SG) from January 2016 to March 2023. Notably, none of the patients were diagnosed with hiatal hernias, nor were any diagnosed intra-operatively with incidental hernias. Furthermore, none of the patients had a history of cardiovascular or thyroid diseases.

### Surgical Technique

The surgical procedure was conducted with the patient positioned in a reverse Trendelenburg position, with an angle of 30°, and the surgeon took position between the legs of the patient. A visual port is introduced after a supra-umbilical 12-mm incision (1–2 cm above the umbilicus), and the pneumoperitoneum is achieved at a maximum of 14 L/min. A 5-mm trocar is inserted at the subxiphoid area liver retraction using a grasper. A 15-mm trocar is introduced at the right upper quadrant, and a 12-mm trocar is inserted at the left upper quadrant. Finally, a 5-mm trocar is introduced at the left subcostal anterior axillary line.

Firstly, the stomach is decompressed via a calibration tube (36F) by the anesthesiologist, which is then pulled above the gastroesophageal junction to facilitate dissection. The omentum is released and ligated from the greater gastric curvature with an energy-based device continuing proximally to the white line of the left crus and distally to the pylorus. Dissection of the hiatus is usually limited to the visualization of the peritoneum above the white line of the left crus, followed by separation of the pad of fat of the anterior cardia.

After the dissection of the greater curvature, the calibrating tube is pushed again and passed through the pylorus by the anesthesiologist. The first linear staplers are placed from the right 15-mm trocar at the right upper quadrant to divide the stomach using a 60-mm green cartridge. The second 60-mm cartridge onward is usually blue or purple according to the thickness of the stomach and is introduced from the left 12-mm trocar. The staple line was reinforced by clips (2016–2020) or continuous sutures using polypropylene 2–0 (2021–2023).

### Anesthesia Protocol

General anesthesia was standardized for all patients. Induction was achieved with 2 µg/kg of fentanyl, 2 mg/kg of propofol until loss of verbal response, and 0.5 mg/kg of atracurium. After 3 min of preoxygenation, intubation was performed. Anesthesia was maintained using isoflurane (MAC 1.2–1.5%) with 50% oxygen in air. Mechanical ventilation was set with a tidal volume of 8 mL/kg and a respiratory rate of 12–15 cycles/min to maintain an end-tidal carbon dioxide tension of 35–40 mmHg and oxygen saturation ≥ 98%. Incremental doses of atracurium were administered every 30 min to sustain muscle relaxation.

To enhance analgesia and minimize postoperative complications, dexamethasone (8 mg), magnesium sulfate (2.5 g IVI), paracetamol (1 g), ketorolac (30 mg IVI), and ondansetron (4 mg) were administered intravenously. Additionally, antibiotic prophylaxis was ensured with 2 g of ceftriaxone IVI. This standardized regimen ensured consistent sedation, analgesia, muscle relaxation, and prevention of postoperative nausea and inflammation across all cases in the study.

### Postoperative Follow-up and Hospital Stay

All patients were sent to the ward for postoperative follow-up. Vital signs were measured immediately postoperatively and every 2 h. Pain control in the ward was managed with IV paracetamol (1 g every 6 h) and ketorolac (30 mg every 8 h). Additionally, IV morphine (3 mg) was administered if the Visual Analogue Scale (VAS) score was greater than or equal to 4. Low molecular weight heparin was given 12 h postoperatively if no intra-operative bleeding was noted. Patients received only pain management medications, intravenous fluids, antiemetics (ondansetron), and pantoprazole. An electrocardiogram (ECG) was performed on all patients, and echocardiography (ECHO) was conducted to assess cardiac recovery. Cardiological follow-up was guided by these results. Patients were followed up weekly during the first postoperative month, then monthly for approximately 1 year, to monitor for any complications.

### Statistical Analysis

Data were analyzed using descriptive statistics in R software version 4.4.1. Continuous variables, including age, BMI, heart rate trends, and time to return to normal heart rate, were summarized using means, standard deviations, and medians with interquartile ranges (where applicable). The Shapiro–Wilk test was used to assess normality. Categorical variables, such as sex, were presented as frequencies.

## Results

The cohort analyzed comprised 8 patients, with an equal distribution of males and females (4 each), and a mean age of 34.88 ± 9.05 years. The mean body mass index (BMI) was recorded at 44.25 ± 3.62. All patients underwent preoperative ECG, and all showed a normal cardiac rhythm. The preoperative mean heart rate was recorded at 80.38 ± 7.3. Intraoperatively, the mean heart rate was determined to be 74 ± 7.91 bpm, which elevated to 79.6 ± 4.8 bpm immediately following surgery. The average total anesthesia duration was 49 ± 7.54 min (Table 1).

**Table 1:** Baseline demographics and perioperative characteristics

Characteristics	*N* (8)
Age (years) Mean ± SD	34.88 ± 9.05
Sex	
Male	4
Female	4
BMI (kg/m^2^) Mean ± SD	44.25 ± 3.62
Co-morbidities HTN	1
Total anesthesia time (minutes) Mean ± SD	49 ± 7.54
Intraoperative heart rate (beats per minute) Mean ± SD	74 ± 7.91
Immediate postoperative heart rate (beats per minute) Mean ± SD	79.6 ± 4.8

**Fig. 1:**
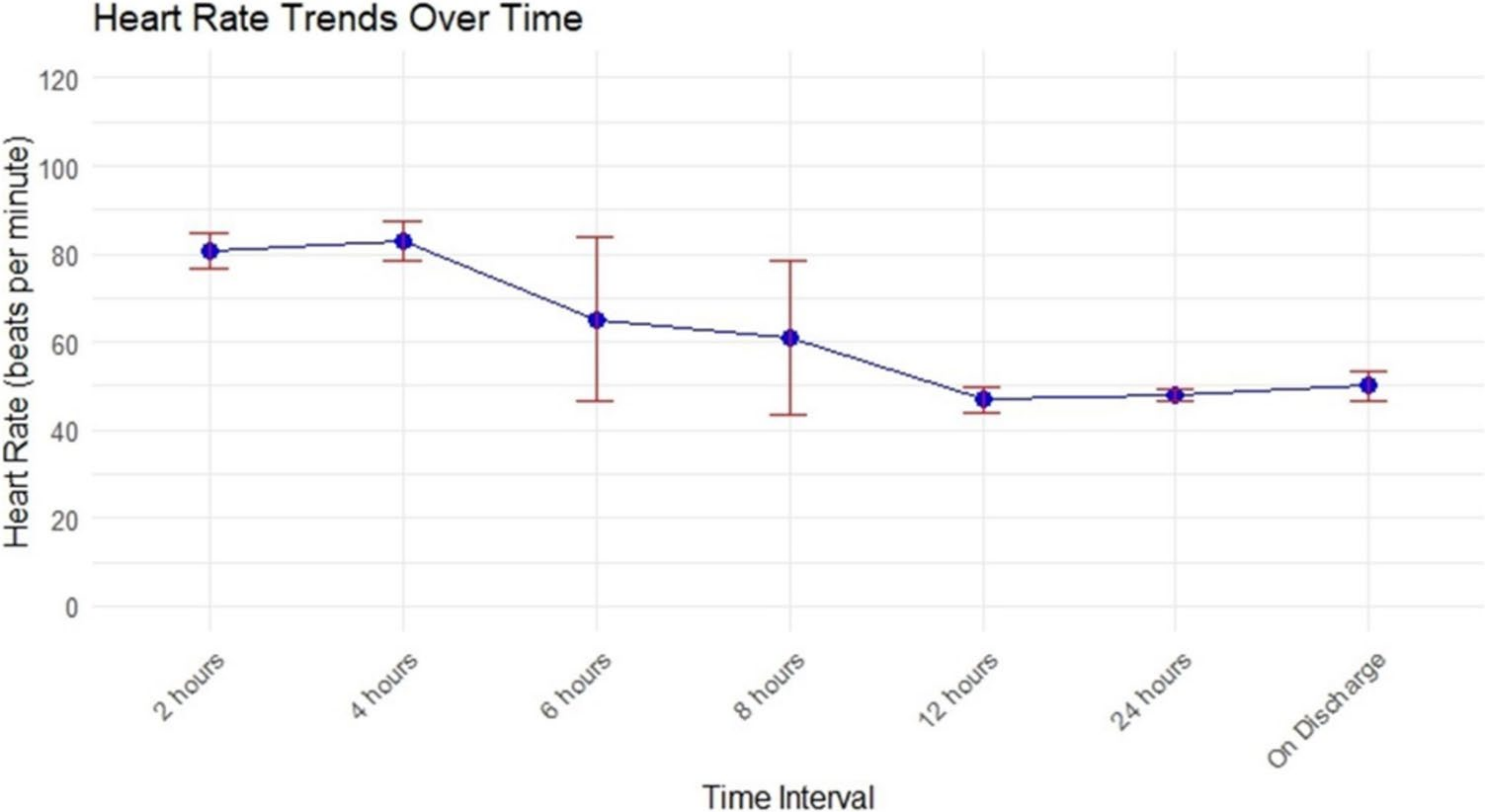
Heart rate trends over time following the surgery

Subsequent postoperative heart rate measurements displayed a gradual decline over time. At the 2-h postoperative mark, the mean heart rate was 80.5 ± 4.04 bpm, with a slight peak at 4 h, recorded at 82.88 ± 4.42 bpm. A distinct downward trend was evident thereafter, culminating in a mean heart rate of 46.88 ± 2.85 bpm by 12 h, and stabilizing at 50 ± 3.17 bpm upon discharge (Tables 2 and 3) (Fig. 1).

**Table 2:** Postoperative heart rate measurements at various time points

Heart rate at various time points (beats per minute)	Mean ± SD
Heart rate after 2 h	80.5 ± 4.03
Heart rate after 4 h	82.9 ± 4.42
Heart rate after 6 h	65.1 ± 18.7
Heart rate after 8 h	60.9 ± 17.6
Heart rate after 12 h	46.9 ± 2.85
Heart rate after 24 h	48 ± 1.31
Heart rate on discharge	50 ± 3.17

**Table 3:** Individual patient demographics and serial heart rate measurements from the preoperative period to 24 h postoperatively

No	Age (years)	Sex	BMI (kg/m^2^)	Hypertension	Pre-operative HR	Intra-operative HR (post-induction)	Immediate postoperative HR	HR at 2 h	HR at 4 h	HR at 6 h	HR at 8 h	HR at 10 h	HR at 12 h	HR at 24 h	HR at discharge
1	35	Male	44	No	81	77	77	80	88	80	55	46	44	49	50
2	28	Female	39	No	75	64	80	88	84	88	80	49	48	47	49
3	42	Female	47	Yes^a^	86	68	75	77	80	44	48	52	49	48	46
4	48	Male	42	No	88	88	79	80	77	80	82	80	47	48	48
5	24	Male	44	No	90	70	88	77	80	43	45	49	43	49	53
6	33	Female	50	No	73	77	85	85	88	80	83	87	44	50	55
7	44	Female	41	No	80	80	74	77	87	49	44	52	50	46	52
8	25	Male	47	No	70	68	79	80	79	57	50	49	50	47	47

*BMI* body mass index (kg/m^2^), *HR* heart rate (beats per minute)

^a^Patient was on valsartan/amlodipine 80/5 mg to control hypertension

**Fig. 2:**
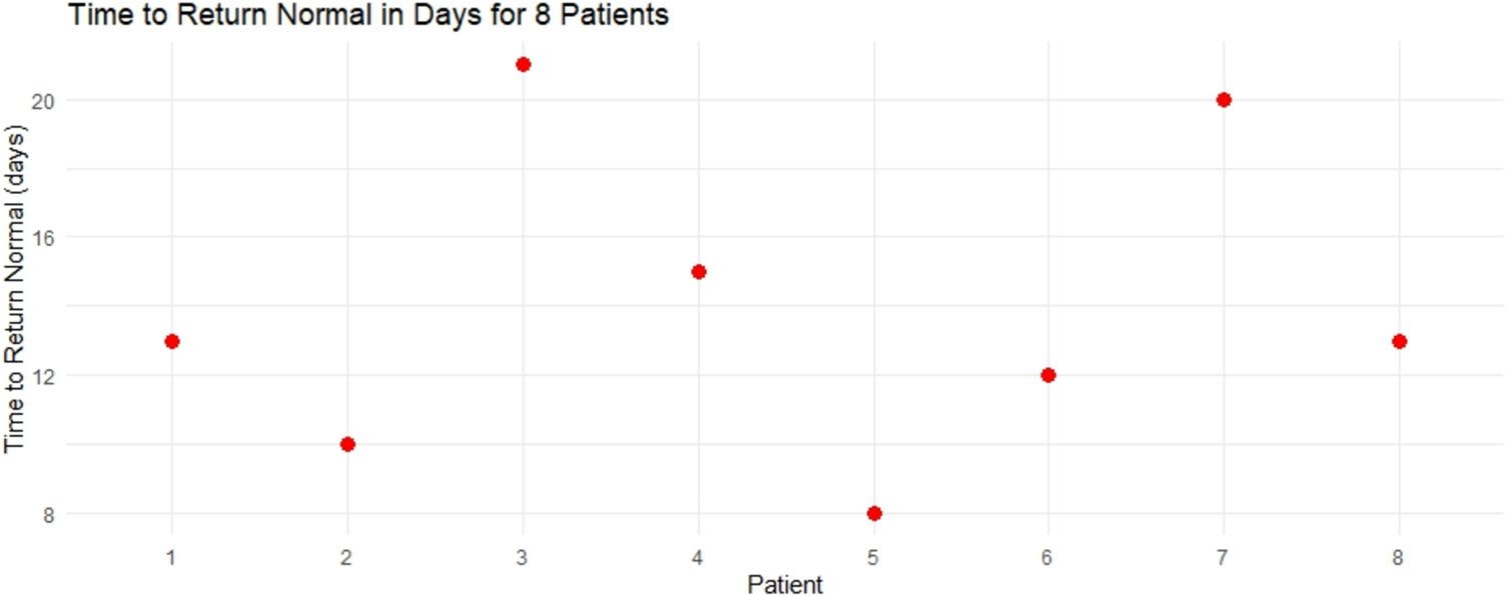
A dot chart visualizes each patient and the time interval to return to a normal heart rate

Postoperative nausea and vomiting (PONV) occurred in three patients, all of whom were successfully managed with 4 mg of intravenous ondansetron. Postoperative pain was managed with intravenous paracetamol and ketorolac in all patients. Only two patients required additional analgesia in the form of intravenous morphine (3 mg), each reporting a VAS score of 5. The length of hospital stays (LOS) had a median duration of 2.5 days (IQR 2–3 days). The average duration to return to baseline heart rate was 14 ± 4.36 days (Fig. 2), while the average follow-up period averaged 14.25 ± 5.47 months (Table 4).

**Table 4:** Characteristics of hospital stay, recovery, and follow-up

Characteristics	*N* (8)
Length of hospital stays in days Median (Q1–Q3)	2.5 (2–3)
Time to return to normal heart rate in days Mean ± SD	14 ± 4.36
Postoperative follow-up period in months Mean ± SD	14.25 ± 5.47

**Fig. 3:**
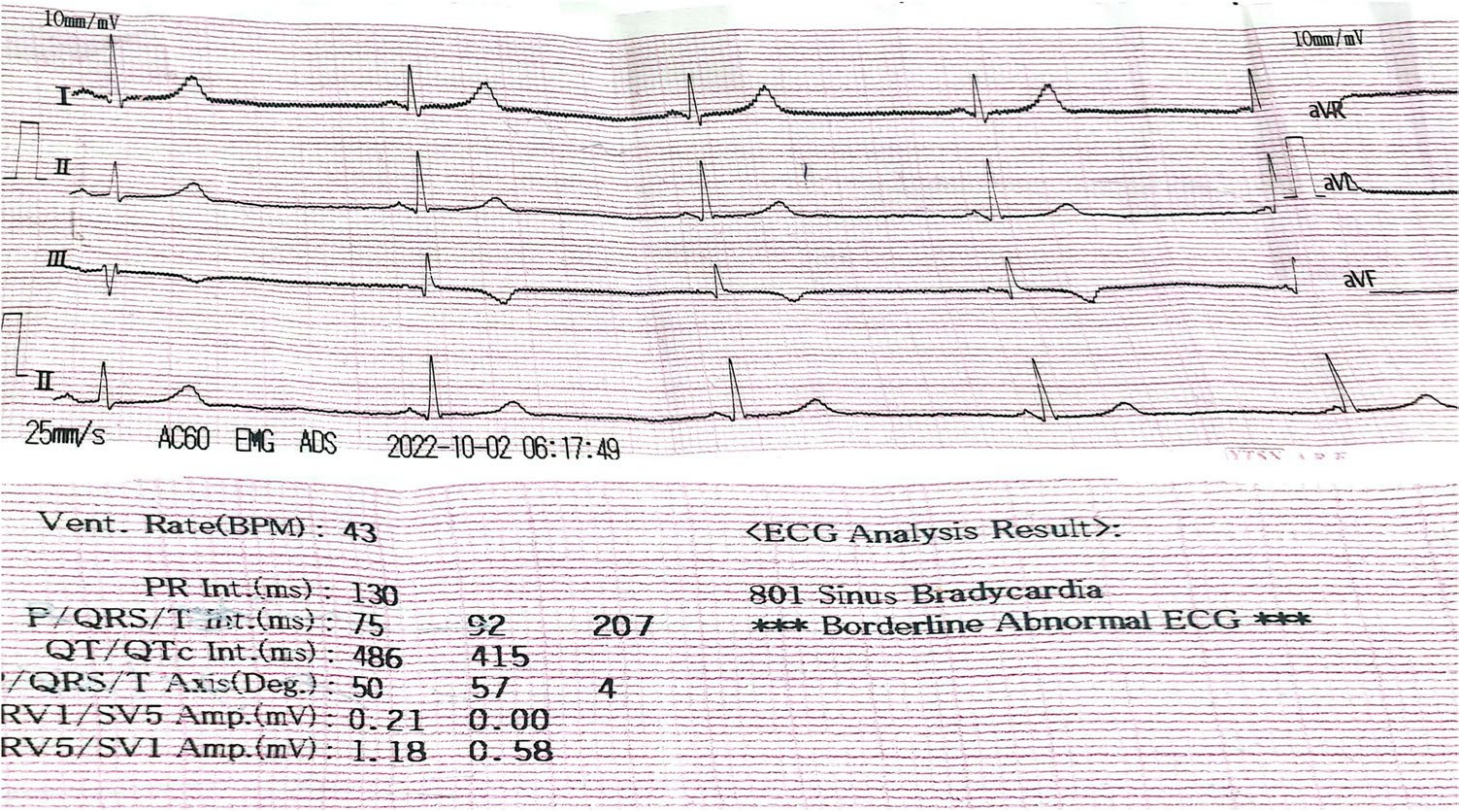
An electrocardiogram showing sinus bradycardia

Notably, heart rate exhibited a pronounced decline after the 4-h postoperative interval, reaching its nadir around the 12-h mark. However, a significant portion of patients experienced a delayed return to baseline heart rate, with an average recovery time of 14 days. Preoperative evaluations, including electrocardiograms (ECGs), displayed no abnormalities, as did echocardiograms (ECHO). Postoperatively, all ECGs and ECHOs continued to show normal findings, except for sinus bradycardia observed in all eight patients (Fig. 3).

### Follow-up

The average follow-up period for these patients was 14.25 ± 5.47 months. Importantly, during this follow-up period, patients did not experience any symptoms related to the bradycardia, such as dizziness or syncope. Furthermore, none of the patients developed any signs of hypoperfusion. Consequently, no interventions, including atropine administration or the placement of temporary pacemakers, were necessary.

## Discussion

Sinus bradycardia may manifest as a late consequence of bariatric surgery, as noted in prior studies. There appears to be a correlation between the degree of body mass index (BMI) reduction and the incidence of sinus bradycardia. A multiple logistic regression analysis of various clinical parameters indicated that the percentage decrease in BMI and the change in heart rate recovery (HRR) following peak BMI reduction were significant predictors of postoperative bradycardia [5, 6]. This phenomenon may be attributed to enhanced vagal tone stemming from decreased BMI, alongside a reduction in adipose tissue that correlates with lower serum leptin levels. These hormonal and physiological changes collectively influence cardiac function, contributing to the development of late-onset bradycardia [5, 6].

Our study also emphasizes the occurrence of early-onset asymptomatic bradycardia during the postoperative period, providing further insights into the postoperative care of patients undergoing bariatric surgery. PONV are common complications linked to general anesthesia and surgical procedures. Research indicates that PONV can affect up to 80% of high-risk patients and approximately 30% of the general population [7].

Additionally, some studies have identified a correlation between PONV and the onset of postoperative bradycardia [8]. During the perioperative phase, bradycardia is frequently attributed to heightened stimulation of the vagus nerve. It is well established that vagal reflexes can provoke sinus bradycardia, elicited by mechanisms such as ocular pressure, carotid sinus stimulation, breath-holding, swallowing, or performing the Valsalva maneuver [9].

In a 2008 study conducted by Billakanty et al. [10], the phenomenon of orthostatic intolerance (OI) following bariatric surgery was examined through tilt table testing among a cohort of 15 patients, although the autonomic function was not assessed. The findings revealed that 53% of patients exhibited a neurocardiogenic response, 20% displayed a dysautonomic response, and another 20% demonstrated symptoms indicative of postural orthostatic tachycardia syndrome (PoTS). The authors concluded that further research is essential to ascertain the true prevalence of OI postbariatric surgery, to delineate which patients are at heightened risk for its development, and to enhance the understanding of the underlying pathophysiological mechanisms involved.

While autonomic dysfunction has been implicated in disturbances in heart rate, including bradycardia and PoTS, most existing literature has focused on delayed autonomic dysfunction following weight loss. Given this context, the potential role of autonomic dysfunction as a causative factor in early postoperative sinus bradycardia warrants further investigation.

Vasovagal syncope (VVS) results from dysregulation of the autonomic nervous system, which affects cardiovascular function, causing bradycardia and hypotension [11]. Loh et al. reported a case of persistent bradycardia (~ 40 bpm) following surgery in a patient who exhibited a neurocardiogenic syncope during tilt table testing [11]. Notably, the heart rate (HR) surged to 120–130 bpm upon initial tilt, contrasting sharply with the resting HR of 40 bpm, suggesting a diagnosis of postural orthostatic tachycardia syndrome (PoTS). Subsequently, the patient was implanted with a pacemaker, leading to the resolution of symptoms; however, the duration of follow-up remains unspecified.

Earlier studies have highlighted the favorable effects of sleeve gastrectomy on the activity of tension-stimulated vagal afferent fibers [12]. This mechanism may elucidate the early satiety and significant weight reduction observed in patients undergoing treatment for morbid obesity [12]. Additionally, it might contribute to the development of postoperative sinus bradycardia due to enhanced vagal stimulation following the procedure [12].

Surgical manipulation of the abdominal cavity can cause transient reductions in arterial blood pressure and alterations in heart rate. Rocco and Vandam et al. reported that patients undergoing abdominal surgeries experienced an average decrease of approximately 20 mmHg in systolic and 6 mmHg in diastolic blood pressure within 8 to 15 pulse beats following manipulation of the upper anterior parietal peritoneum, liver retraction, or packing [13]. These changes were attributed to tissue deformation, which increases vagal tone and decreases cardiac contractility and output. Notably, bradycardia occurred simultaneously in nearly half of the patients, highlighting the interplay between these factors [13].

To build upon the findings of this study and test the proposed hypothesis, future research should focus on comparative studies between SG patients who develop postoperative bradycardia and those who do not, aiming to identify predictive factors and modifiable risks associated with this complication. Advanced cardiac diagnostics, such as heart rate variability (HRV) analysis and autonomic function testing, should be incorporated to explore the role of vagal tone and other autonomic factors in the development of early bradycardia.

This study is limited by its small sample size of eight patients, which restricts the generalizability of its findings. Furthermore, incomplete cardiovascular data hampers a more comprehensive analysis of the observed phenomenon. Despite these limitations, our findings underscore the need to recognize early postoperative sinus bradycardia as a potential complication of SG. This observation emphasizes the importance of heightened awareness and close monitoring, as it may necessitate timely interventions. Further research should aim to establish clinical guidelines for managing this phenomenon, ensuring it does not progress to symptomatic or critical arrhythmias.

## Data Availability

No datasets were generated or analysed during the current study.

## Abbreviations

BMI: Body mass index
ECG: Electrocardiogram
ECHO: Echocardiogram
HR: Heart rate
HRR: Heart rate recovery
HRV: Heart rate variability
IV: Intravenous
NS: Normal saline
PONV: Postoperative nausea and vomiting
PoTS: Postural orthostatic tachycardia syndrome
SG: Sleeve gastrectomy
VVS: Vasovagal syncope
